# Supplementary figures and images for: Single-Cell Transcriptomics Analysis Reveals a Cell Atlas and Cell Communication in Yak Ovary
Source: Int J Mol Sci. 2023 Jan 17;24(3):1839. doi: 10.3390/ijms24031839 (PMC9915757; doi:10.3390/ijms24031839)

**nFeature\_RNA**

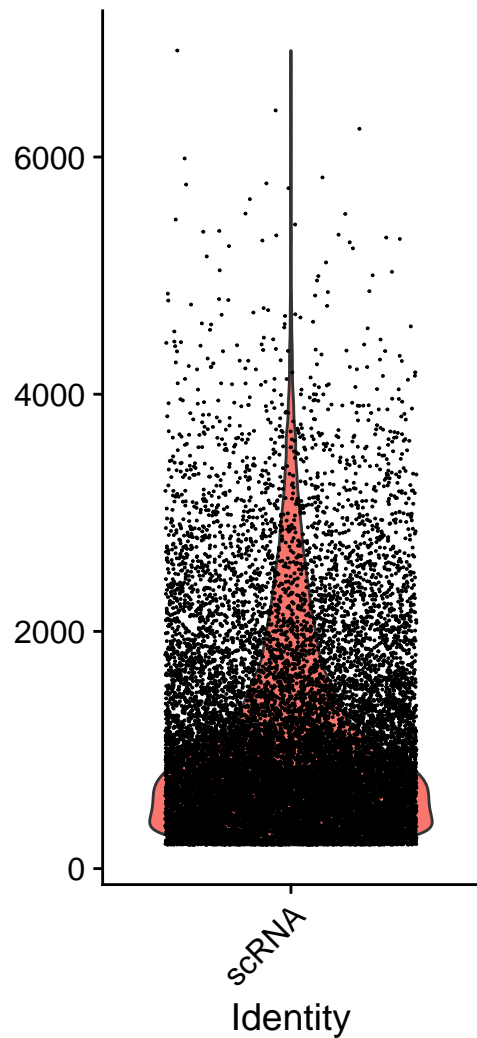

**nCount\_RNA**

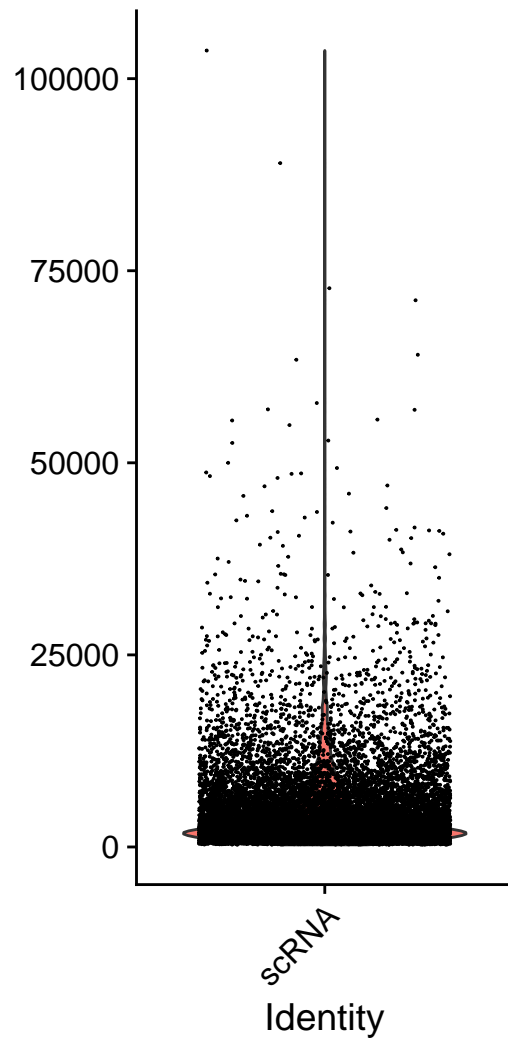

**percent.HB**

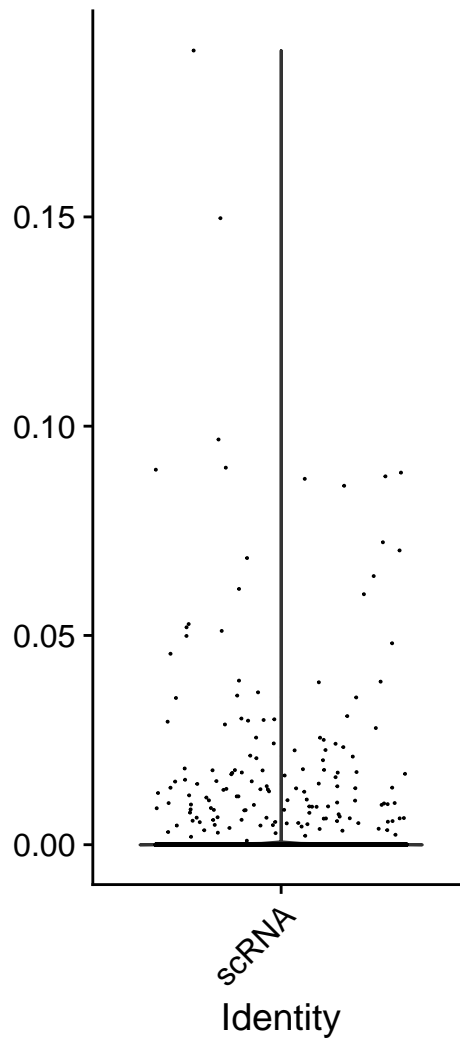

**percent.MT**

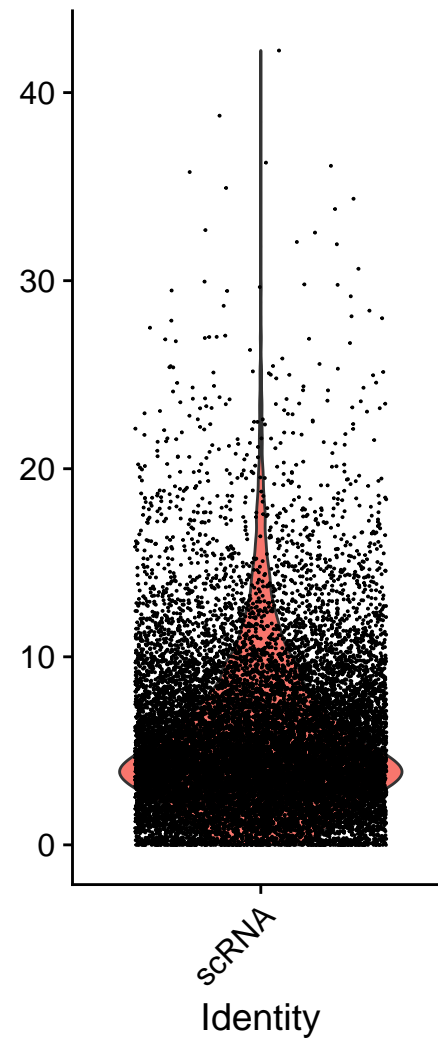

**percent.Ribosome**

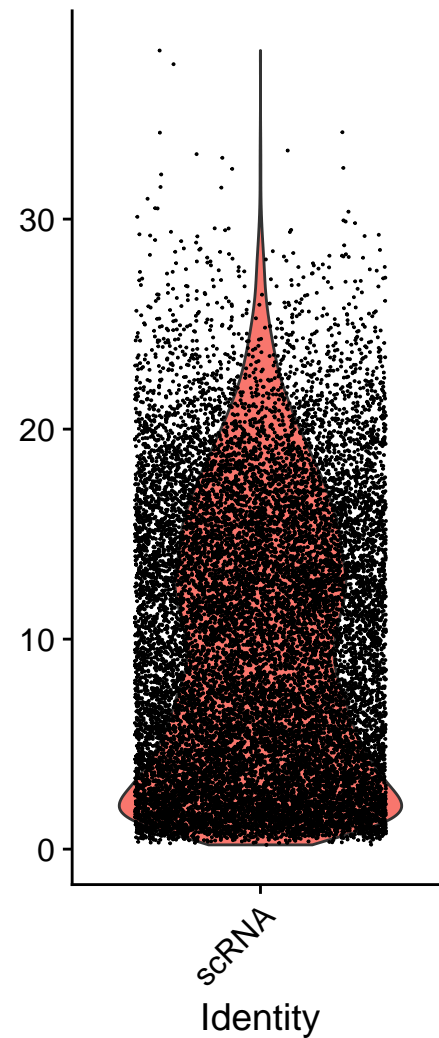

Supplement: Supplementary file 1 [file ijms-24-01839-s001.zip › Supplementary_Figure_S1_Percentages_of_nFeature_nCount_HB_MT_Ribosome.pdf]

# SingleR

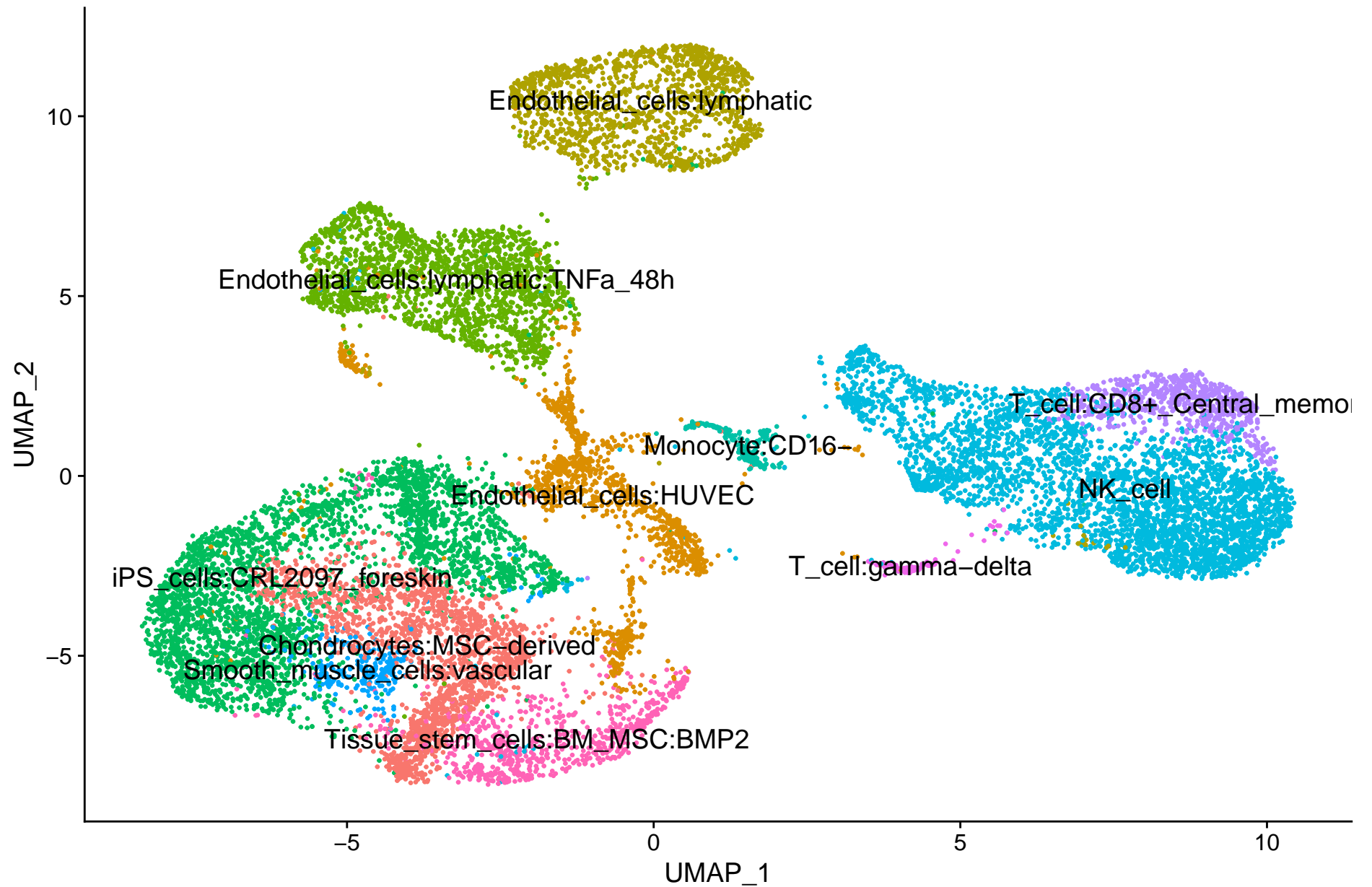

Supplement: Supplementary file 1 [file ijms-24-01839-s001.zip › Supplementary_Figure_S2_Cluster_annotation_by_singleR.pdf]
